# Models of *Diabrotica* Populations: Demography, Population Genetics, Geographic Spread, and Management

**DOI:** 10.3390/insects11100712

**Published:** 2020-10-17

**Authors:** David W. Onstad, Michael A. Caprio, Zaiqi Pan

**Affiliations:** 1 Corteva Agriscience, Johnston, IA 50131, USA; 2 Department of Biochemistry, Molecular Biology, Entomology and Plant Pathology, Mississippi State University, Mississippi State, MS 39762, USA; mac24@msstate.edu; 3 Corteva Agriscience, Chestnut Run Plaza 735/4175-3, 974 Centre Rd, Wilmington, DE 19805, USA; zaiqi.pan@corteva.com

**Keywords:** *Diabrotica barberi*, *Diabrotica virgifera virgifera*, insect resistance management, population dynamics

## Abstract

**Simple Summary:**

Two beetles that are serious pests of maize, *Diabrotica virgifera virgifera* and *Diabrotica barberi*, have caused problems for farmers in the USA and Europe for many years. Because both species have developed resistance to several management tactics, including insecticides and crop rotation, mathematical modeling has been used to evaluate their life cycles for weaknesses and new tactics for value. This review highlights lessons learned from the past 35 years. Some models have focused on the probability of the beetles spreading across regions. Other models have been developed to estimate the risk of the evolution of resistance. These models are thoroughly reviewed with respect to the biological attributes incorporated in these models and the impact of those attributes on the evolution of resistance.

**Abstract:**

Both *Diabrotica virgifera virgifera* LeConte and *D. barberi* Smith and Lawrence are among the most damaging insects impacting corn in North America. *D. virgifera virgifera* has also invaded Europe and has become an important pest in that region. Computer models have become an important tool for understanding the impact and spread of these important pests. Over the past 30 years, over 40 models have been published related to these pests. The focus of these models range from occupancy models (particularly for Europe), impact of climate change, range expansion, economics of pest management, phenology, to the evolution of resistance to toxins and crop rotation. All of these models share characteristics. We elaborate on the methods in which modelers have incorporated the biology of these pests, including density-dependence, movement, fecundity and overwintering mortality. We discuss the utility of both spatially-explicit, complex models and spatially-implicit, generational models and where each might be appropriate. We review resistance models that either explain past evolution to crop rotation, insecticides or insecticidal traits or attempt to predict the consequences of resistance management strategies.

## 1. Introduction

Mathematical modeling allows one to explore scenarios and conditions that would be difficult to study with experiments. Time horizons can be much longer and spatial scales much larger. Models can be used to explain the past or predict the future. They can be used solely for biological purposes, or modeling can help us improve management and evaluate economics. In this review, we focus on the history of modeling populations of *Diabrotica* species with an emphasis on biology, but with consideration of management. 

*Diabrotica* (Coleoptera: Chrysomelidae) are important pests of corn (*Zea mays*) in the Americas and, over the past few decades, also in Europe [1]. The larval stage feeds on plant roots, making data collection more difficult compared to species with larvae that feed above ground. An interesting aspect of *Diabrotica barberi* Smith and Lawrence (DB) and *Diabrotica virgifera virgifera* LeConte (DVV) in North America, with regard to both management and population biology, is their adaptation to crop rotation [2].

We present a narrative that describes the purpose of each model and highlights the unique contributions made by modelers as they create new models that often, as much as science does, build upon the experimental and modeling work of earlier scientists. We have chosen to ignore or de-emphasize conclusions drawn by authors concerning management, because these are likely specific to a given product and a *Diabrotica* phenotype that are not likely to be encountered by modelers in the future [3]. The lessons about how to create models are what we hope to share with the next generation of modelers. 

One of the earliest models of *Diabrotica* addressed whether adulticide applications could reduce populations sufficiently so that subsequent larval damage would be ameliorated [4]. This early model illustrated the important interplay between models and field research. Although the paradigm is often assumed to be field research followed by model development, this model also illustrated the alternative paradigm of model development (first to identify data gaps and weaknesses) followed by prioritization of field research to address those gaps. In this scenario, the models identify, through processes such as sensitivity analysis or risk assessments, not just data gaps, but the data gaps that are most important to answer the particular questions being addressed. 

This review is divided into several sections. First, we review models that emphasized the population biology of DVV. Then, we do the same for models of DB. Next, we review models simulating evolution of resistance to crop rotation. Fourth, we consider models simulating evolution of resistance to insecticides and insecticidal traits. Fifth, we review models of geographic spread of DVV. Finally, we draw conclusions in the discussion.

The models reviewed here consider environmental space in a variety of ways. Spatially implicit models are usually the default choice because they are easier to create and analyze when modeling populations. However, when the goal of the project requires, or knowledge of reality motivates, we often model populations moving and interacting in explicitly defined space, such as when we need to study geographic spread of pests or dispersal across heterogeneous landscapes. In these cases, spatially explicit models should be built, if supporting data are available.

## 2. Fundamental Models of *Diabrotica virgifera virgifera*

Many different types of models have been useful in addressing a variety of questions regarding *Diabrotica* biology. The parameters and functional forms of the parameters vary with the questions being addressed by the models. Models that address impacts of climate change on *Diabrotica* distribution will likely include temperature dependent functional forms for many parameters, while models focusing on evolution are less likely to do so. Before construction of any model it is key to understand how the model is to be used and the questions or hypotheses to be addressed. 

To date, only one complete age-specific life table has been published for DVV [5]. There are several observations from this study that may be useful for modeling DVV. The first is that while the greatest single mortality factor in the life table is related to the establishment of first instars, there is little variance between years in this estimate. The greatest impacts on differences in growth rates between years were the overwintering egg mortality rate and mortality of second and third instars. Secondly, there were large differences between potential fecundity, measured as the fecundity of adults held under ideal laboratory conditions, compared to realized fecundity, the fecundity measured from similar adults held under field conditions in cages. The realized fecundity ranged from 13.1% to 16.1% of the potential fecundity (estimated at 353 eggs/female rather than the maximal observed egg production). Third, at least in southern Hungary, the natural enemy complex and pathogens had very little impact on growth rates. This may be related to the relatively recent introduction of DVV in Hungary. Ultimately, all of the life tables developed in Toepfer and Kuhlman [5] had growth rates less than one, suggesting the population sizes were decreasing. Use of the absolute parameter estimates from these tables may not be warranted when modeling areas where DVV commonly achieves pest status, but the overall pattern of life table parameters may still be useful.

### 2.1. Density Dependent Mortality

DVV has long been known to have decreased survival when densities of larvae are high [6,7,8,9,10,11,12]. Elliot and Hein [13] incorporated density dependence into an early population dynamics model of western corn rootworm, focusing on interactions with the corn plant growth stage. They suggested density-dependent mortality was negligible below 466 eggs/m^2^. Genetics was not considered in this model. Onstad et al. [14] developed the first DVV resistance model that incorporated density dependence. Based on Branson and Sutter [6], Gray and Tollefson [15] and Elliot et al. [10], Onstad et al. [14] modeled density-dependent survivorship as 1/(1 + 2.42 × 10^0.7^), where E is the egg density in millions/ha. The density-dependent survivorship in this model of resistance to transgenic plants occurred after mortality due to the toxins. This same equation was adopted in a model by Storer [16]. Note that because the denominator approaches 1 as the egg density decreases, this equation assumes that other density-independent survival factors are incorporated in other equations of the model. Crowder and Onstad [17], further developed a model incorporating rotation resistant DVV [18] using 1/(2.59 + 1.29 × 10^0.88^) for density-dependent survival. Caprio et al. [19] split the survivorship curve into two sections (piece-wise regression), assuming that density dependent-survival was 1.0 if egg densities were below 288 million eggs/4.05 ha (ca. 960 eggs/plant). Above this cutoff, density-dependent survival was calculated as the maximum of 0 or 0.012 × 10^(×10^−0.004^)^, where E is the egg density in millions/4.05 ha. 

Ultimately, while all these approaches to density-dependent survival vary in specifics, it is perhaps most important that density-dependence is considered, particularly in multi-patch models where densities between patch types can vary [20,21,22]. Martinez et al. [23] did note that the actual form of density-dependence can impact rates of resistance evolution. Haridas et al. [24] modeled the interaction of the mean and variance of soil temperature with density dependent and independent mortality utilizing larval survivorship data from Hibbard et al. [12]. They suggested these interactions could result in populations that exhibit population size equilibrium, cycling or extinction. Onstad et al. [11] summarize many studies on density dependent survivorship in *Diabrotica*. 

### 2.2. Oviposition and Fecundity

DVV females go through an extended, circa 13-day, preovipositional period after emergence. Oviposition rates are high after this period, declining as females age [25]. Elliott and Hein [13] used a maximal rate of 310 eggs/female [26], modified by the plant growth stage, insect age and temperature. Onstad and collaborators [11,14,17,18,27,28,29] used a fecundity rate of 440 eggs/female (or 220 female eggs/female). Storer [16] assumed females laid a maximal rate of 23 eggs/day for a total female fecundity of 118 eggs/female. This was further modified by the age and genotype of the female and her mate. Caprio et al. [19] used an oviposition rate of 29 eggs/day for a 28 day stage following a 13 day pre-ovipositional period. Older females produced 7.5 eggs/day. Based on the daily adult mortality rates in the life tables, this resulted in a net reproductive rate of 11.35 female adults produced on average per female (ignoring overwintering egg mortality which averaged 50%). Given the average survivorship to the adult stage of 3.0%, the mean number of female eggs oviposited by an average female was 378.3. Pan et al. [30] assumed a maximal rate of 356 eggs/female, though actual fecundity depended on genotype, natal habitat and dominance of resistance alleles.

To more accurately model oviposition over the season, Onstad et al. [14] accounted for adult feeding on corn and noncorn plants and aging of adults when calculating adult mortality. Based on data collected by Elliott et al. [31], Onstad et al. [14] created a function for nutrition-based mortality of adults. As the corn crop matures beyond the early reproductive stages, adult mortality increases. 

### 2.3. Adult Dispersal

Dispersal can be measured in several different ways depending on the form of the model. Some models use a generational time-step, others use a daily time-step, e.g., Crowder and Onstad [17], Crowder et al. [28]. Some models are spatially explicit, that is there is a specific ordering and structure to patches (fields) so some patches are closer while others are more distant (concepts of distance, dispersal kernels and spread become important) [14,16,19,29,30,32,33,34,35,36]. Other models are spatially implicit and model populations with no spatial structure and movement is represented as an exchange of individuals between these populations without regards to distance [37,38,39,40,41,42]. Dispersal estimates for DVV are also complicated by the fact that several flight mill studies indicated both a trivial flight and sustained flight behavior [43,44,45]. Different models may focus on different aspects of these two behaviors. Recent studies using mark-recapture techniques in response to the introduction of DVV into Europe have provided additional data that might be useful in modeling movement of DVV [46,47,48]. Estimates from all the above studies have suggested that between 24–38% of the adult DVV population engages in density-independent, long-distance dispersal. Higher densities may increase the proportion of beetles engaging in long distance dispersal [48], although Naranjo [49] found a negative relationship between larval density and sustained flight behavior in a flight mill study.

Elliott & Hein [13] used a daily migration rate of 15%/day for females aged 5–10 days and a rate of 7.5% for similar aged males. This was a model of a single population, so emigration of adults was treated mathematically as mortality. Onstad et al. [14] used a daily between field dispersal rate of 2% for females and 0.5% for males. Storer [16] varied the daily dispersal rate depending on crop and crop phenology. The daily dispersal rates were 5%, 2.5% and 15% from preflowering, flowering and mature stage corn respectively. Due to the nature of the dispersal kernel not all of these dispersal events resulted in movement between fields. Crowder et al. [28] assumed a panmictic distribution over the fields they simulated, at least for individuals not resistant to crop rotation. Crowder and Onstad [17], using a generational time step model, simulated a similar panmictic movement pattern but with the addition of some male dispersal prior to mating (all females mated in their natal field). They noted “that the results of the daily time step and generational time step models were similar and that the complexity involving dispersal, mating, emergence, and oviposition needed to develop the daily model did not affect the results as significantly as other assumptions”. Nonetheless, should one wish to approach a problem tied to a daily timestep, such as developmental delay of larvae, a daily timestep model might allow elucidation of that factor with fewer abstractions and more easily measured assumptions than a simpler generational model. Caprio et al. [19] only simulated long distance dispersal, assuming populations within patches/fields were panmictic. They used a dispersal rate of 1.13%/day for preovipositional adults (15% of all adults dispersed over the stage) and 0.2%/day for older adults (resulting in an additional 7.4% of adults engaging in long distance dispersal), for a total of 22.4% of all adults engaging in long distance dispersal. A different measure of dispersal would be the location of where adults placed their eggs, combining age-specific dispersal, mortality and fecundity rates. Caprio et al. [19] estimated that 83.7% of the eggs in their model were oviposited in the natal field. As with most multi-patch models, movement of mated females actually results in the movement of two genomes, that of the female and the male with which she had mated. Because DVV females only mate once or twice, this can result in greater gene flow than might be observed in species that mate frequently. 

Other models have focused on the implications of fine-scale movement for local population dynamics. Pan et al. [30] focused on within field dispersal using estimates from mark-recapture studies. They simulated 80 ha corn fields that were further subdivided into a 10 × 8 grid of 1 ha cells. Movement was simulated with an exponential distribution where the probability of moving a given distance is a function of the mean daily dispersal distance, assumed to be 15 m/d for both males and females) and the direction was drawn from a uniform circular distribution. Males dispersed upon emergence while females did not disperse until mated. The goal of this model was to more realistically describe the effects of local dispersal on insect resistance managenent (IRM). Caprio and Glaser [35] focused on local dispersal within fields using a simulation system of 0.09 ha (30 × 30 m) patches. They assumed that 55% of beetles moved per day and moved an average of 29 m/day. This value is similar to the value used in Pan et al. [30] but only measures the distance moved by the 55% of the beetles that engaged in movement. Because beetles were assigned a random position in the 30 × 30 m block and movement was in a random direction, not every movement led to a change in the block the insect was assigned to. The distance moved for each movement was based directly on random draws from binned movement data [50], and therefore was a direct translation of field measurements. These two models demonstrated that incorporating fine-scale measurements of movement with mating, fecundity, oviposition and mortality can alter the evolutionary trajectory of local populations compared to simulations that assume random mating and oviposition within patches or fields.

### 2.4. Overwintering Mortality

Overwintering mortality is an important factor in the yearly univoltine dynamics of DVV. Most authors have used a value in the range of 50% mortality during the overwintering phase [11,14,17,28,29]. Caprio et al. [19] also used 50% overwintering mortality but in an attempt to simulate yearly differences in population densities, that value was varied as a random draw from a normal distribution with a mean of 0.5 and a standard deviation of 0.25, with limits bounded by (0.05,1.0) Storer [16] does not explicitly state an overwintering mortality, but does use a density-independent egg survivorship of 0.05.

## 3. Fundamental Models of *Diabrotica barberi*

DB is well-known for variation in length of diapause in natural populations. Instead of diapause occurring only over one winter, mutants maintain diapause over two to three winters (and time in between). When crop rotation became a common practice, prolonged or extended diapause became more prevalent. Adults feed on many host plants besides corn, and may move long distances from corn fields [51].

In a series of papers, Naranjo described the creation and analysis of a relatively detailed model of DB adult dynamics over time in a single field of corn (*Zea mays*). In an excellent paper, Naranjo and Sawyer [52] described their core submodel for DB reproduction. Their model had temperature-dependent maturation through the adult stage and time and age-dependent oviposition. They used temperature to determine mean rate of maturation (physiological time) and included a temperature-independent distributed delay to determine the proportion of each cohort developing to the next stage. The stages modeled included the pre-reproductive, reproductive and post-reproductive stages of the female population. Mean rates of maturation were nonlinear functions of temperature. The model kept track of the number of individuals in each cohort and their physiological age. They calibrated the model and all of its equations by fitting curves to maturation and oviposition data that they collected. An hourly time scale was used to calculate physiological time and a daily time step was used to determine oviposition and cohort transition to the next life stage. The model was based on studies performed at constant temperature. Naranjo and Sawyer [52] validated the model against independent data collected at constant and fluctuating temperatures. Their model fit the observations well. A sensitivity analysis demonstrated that results were very sensitive to errors and variability in input temperatures. 

Naranjo and Sawyer [53] expanded their core submodel of adulthood to quantify and validate the effects of crop phenology on adult populations and oviposition and to study the role of beetle dispersal in the dynamics within a single cornfield. They added a variable for density of adult males and modeled three growth stages of corn. The time steps remained the same as for the original core submodel [52]. Adult mortality was calculated from a function of nutrient availability in the corn crop. All functions were fit to data collected by Naranjo. The flowering stage of corn provides the necessary nutrients and influences not only mortality but also adult dispersal as described by Naranjo and Sawyer [52]. The model results were extensively tested against independent field data collected by Naranjo. Naranjo and Sawyer [53] urged entomologists to measure DB dispersal and feeding behavior (the weakest parts of their model) to improve the model and management.

Naranjo and Sawyer [54] performed extensive sensitivity analyses on their expanded model. One goal was to examine the relationship between adult abundance, oviposition and corn phenology. Another goal was to formalize hypothetical improvements in DB management based on adult sampling. Model results were very sensitive to changes in age-specific oviposition rates and to factors influencing the timing and duration of the flowering period of corn. Model results were moderately sensitive to changes in the timing of female emergence, rates of pre-reproductive female dispersal at peak flower, fecundity and maturation rates during reproduction. 

Mitchell and Riedell [55] expanded on the work of Naranjo and Sawyer [53] in two major ways. First, they emphasized the variable and stochastic nature of temperature effects on DB maturation. Second, they included maturation and mortality of the immature stages (egg, larvae, and pupae). They incorporated stochastic density-dependent mortality of larvae based on field data. They believed that adult movement affects adult mortality because of feeding throughout the landscape not just in a single field. Daily rate of adult mortality depends on the availability of pollinating corn in all the fields in a region. Mitchell and Riedell [55] used the Naranjo and Sawyer [53] pollination model to calculate the proportion of plants flowering in the field of emergence. They expanded this with a submodel that calculates the availability of pollinating corn in all fields in the landscape. They fit a regression model to data on fields of corn and then converted to amount of plants pollinating. 

Mitchell and Riedell [55] compared their results to a variety of field-data sets. Because of nonlinear equations, they concluded that realistic, variable temperatures produce better simulations of population dynamics compared to deterministic simulations using mean temperatures. A sensitivity analysis indicated that model results were extremely sensitive to some of the parameters defining crop phenology and its effects on adult mortality. 

Mitchell and Onstad [56] created a generational time-step model of the entire life cycle that accounted for prolonged diapause in a population of DB in a landscape consisting of various patches of corn, soybean and wheat with and without rotation of corn. The purpose of the modeling was to study the evolution of resistance to transgenic insecticidal corn in this population. They calibrated a density-dependent larval survival function using field data [57]. Because Naranjo and Sawyer [53] concluded that their approach to calculating fecundity underestimated realistic values, Mitchell and Onstad [56] used the data of Boetel and Fuller [58] to choose a relatively high constant mean fecundity. Although previous models had emphasized the role of pollination and corn phenology in the movement of adults, Mitchell and Onstad [56] simplified the model and assumed eggs are uniformly distributed over all corn patches. They also assumed that some adults mate within their natal patch and a larger proportion mate randomly throughout the corn landscape.

Mitchell and Onstad [56] emphasized the influence of extended diapause on the evolution of resistance to transgenic insecticidal corn. Based on data, they assumed that the total hatch rate for eggs was 60%. The standard simulations either had no extended diapause (60% hatch after first winter) or distributed this total as 40% hatch for eggs after one winter, 15% after two winters, and 5% after three winters. In their extensive analysis, they varied and increased the proportion hatching after two or three winters but did not model evolution during a simulation. Mitchell and Onstad [56] concluded that extended diapause in DB has two offsetting effects on evolution of resistance to insecticidal corn. First, extended diapause injects older alleles with lower resistance allele frequencies into the breeding population, which slows resistance. Second, extended diapause speeds the population’s recovery from perturbations (reduces the undercompensating, density-dependent larval survival) which accelerates resistance.

## 4. Modeling Evolution of Resistance to Crop Rotation

Alternating corn with another crop every other year was a very good design for managing *Diabrotica* in North America. However, DB and DVV adapted to corn-soybean rotation schedules in the United States. As noted above, prolonged or extended diapause in the egg stage became more prevalent in DB populations. DVV evolved oviposition behaviors that allowed mutants to lay eggs not only in cornfields but also in most other crops. Essentially, DVV lost its fidelity to corn as a location for oviposition because of the fitness advantages within a two-year crop rotation schedule [59]. 

The only model to consider rotation-resistance for DB is described above [56], but it does not explain or predict the evolution of resistance to crop rotation. A series of models produced by the Onstad lab considered the evolution of rotation-resistance by DVV and its management [59].

Onstad et al. [60] modeled the evolution of behavioral resistance by DVV to crop rotation that first occurred in Illinois in the USA. It was a simple, frequency-based (population genetics) model with a generational (1 year) time step. The model consisted of four plant patches: corn, rotated corn, soybean, and extra noncorn plants. The proportion of soybean in the landscape equaled the proportion of rotated corn. They assumed one gene for resistance with three alleles. Beetle phenotypes were corn-only wild-type individuals, soybean specialists, and those ovipositing in all vegetation. Onstad et al. [60] also defined the complex behaviors of genotypes with additive alleles. Mating was random in each patch, but nonrandom in the landscape. Movement and distribution of eggs was determined by the phenotype of the beetle and the proportional areas of the host plants in the landscape. All larvae hatching in noncorn patches including soybean die. Onstad et al. [60] determined selection by accounting for relative fitness (0–1) for each of the twelve genotypes. Relative fitness was calculated from logical formulas for relative fecundity and larval survival.

Onstad et al. [60] tested the model results against the first observation of resistance, which occurred about 16 years after the beetle invaded Illinois. Results showed that the greater the level of corn-soybean rotation in the landscape, the faster the evolution of the ovipositing generalists. Several parameter sets allowed the model results to match reality. The conclusion that this phenotype is the one that evolved was supported by the observation that beetles can be found in crops other than soybean. Model results demonstrated that the soybean specialist would have more difficulty evolving than the ovipositing generalist. Therefore, Onstad et al. [60] concluded that the soybean specialist did not evolve in Illinois.

Onstad et al. [18] updated and extended the model of Onstad et al. [60] based on data collected after the first publication. They modeled fecundity as total eggs per female beetle and incorporated density-dependent larval survival that is applied once per generation after overwintering and insecticidal mortality. Onstad et al. [18] used the improved, density-based model to evaluate seven management strategies including the typical two-year crop rotation. The six alternative system designs included host-plant resistance by repellency or insecticidal corn and unusual cropping schedules such as a three-year rotation or less rotation and more continuously planted corn.

Crowder et al. [28] and Crowder and Onstad [17] extended the models of Onstad et al. [14,18] to include more details about transgenic insecticidal corn or crop rotation. They analyzed multiple scenarios: both technologies/practices in areas with rotation-resistance or areas without rotation-resistance, and only one of the technologies/practices in each area. Crowder et al. [28] increased the proportional dispersal away from natal patches to allow results to match published data. Based on results of Onstad et al. [14], they simplified the mating behavior of the beetles. Crowder et al. [28] used the daily time-step model to study how results compared to observations of field emergence and apparent early mortality of adults in transgenic insecticidal cornfields. Crowder and Onstad [17] provided an interesting comparison of results from both the daily and generational models.

## 5. Modeling Evolution of Resistance to Insecticides and Insecticidal Traits

This section focuses on models that either explained past evolution of DVV to insecticides or insecticidal traits or attempted to predict the consequences of IRM strategies. Some models are hypothetical, while others are applied to particular insecticides or corn traits. The spatial aspects of the models and their complexity are determined by the goals of the projects (Table 1). We describe the processes that influenced model results, particularly expression of resistance, adult dispersal, and density-dependent survival. All the models described below assume that insect resistance is conferred by an autosomal, di-allelic major gene for each insecticide or insecticidal trait. Unless noted otherwise, resistance to each toxin is complete (100% survival by homozygous resistant beetles). In the models, initial resistance allele frequency is usually 0.0001–0.001. 

When refuge of non-Bt corn is deployed as a seed blend (blended refuge) with Bt corn in the same field, larval behavior and related mortality factors are usually included in models. Although much attention is placed on movement from plant to plant, the most important question is Does survival, and therefore selection, differ between genotypes because of movement? Thus, movement per se may not be significant if heterozygotes do not have an advantage due to feeding and movement amongst refuge and insecticidal plants. Onstad [61] and Pan et al. [30] assumed that (1) only a fraction of the young larvae move and (2) movement occurs once per larval stage, and (3) most feeding by DVV larvae occurs after the one movement. Only minor mortality occurs due to tasting Bt corn before dispersal away from those plants in their models. Caprio and Glaser [35] modeled larval movement in their frequency-based deterministic model. They permitted daily chances for movement for all larvae. Thus, some larvae will move a few times and others will move often. Some will move later and others earlier in the larval stage. 

Onstad et al. [14] created a deterministic simulation model of the population dynamics and genetics of DVV for a landscape of corn, soybean, and other crops. The model was published before any *Bt* transgenic rootworm-resistant corn was commercialized and was used to evaluate potential resistance management plans for transgenic corn. Results helped to identify research priorities and assisted those creating resistance management plans. The model simulated a crop landscape with continuous corn, rotated corn, soybean, and an extra noncorn crop. The model used a daily time step and was spatially implicit with most DVV biology of egg, larva, pupa and adult stages. Adult dispersal between different crops was also simulated in the model. The model assumed that no first instar DVV moved away from transgenic corn roots as only block and strip refuge was simulated. The density-dependent survival of larvae occurred after overwintering survival and survival due to conventional and transgenic plant toxins. This model was unique in its incorporation of mating complexity. Mating amongst DVV emerging in block refuge and fields of Bt corn is a concern as female beetles fly short distances before mating [62]. Based on lab data, the model assumed that female adults could mate in a second phase after the first oviposition period. Furthermore, Onstad et al. [14] calculated the probability of a female mating based on male density and search capability. They evaluated the sensitivity of results to male density and searching capability in blocks of corn. The standard search area was 100 m^2^. Changing the area between 30 m^2^ and 170 m^2^ searched by a male each day for a mate had insignificant effect. Therefore, later models assumed mating would not be a concern and assumed that almost all realistic male densities could guarantee all females would be mated. 

Onstad et al. [14] found the gene expression in DVV and toxin dose in the corn plant were the two most crucial factors affecting resistance development. The model results were not very sensitive to the refuge size between 5% and 30%. Because this might be due to density-dependent mortality in block refuges, the authors explored density-dependent survival in their future papers.

Storer [16] created a stochastic, spatially explicit computer model to simulate the adaptation by DVV to insecticidal traits in corn. The model included functions for crop development, egg and larval mortality, adult emergence, mating, egg laying, mortality and dispersal, and alternative methods of DVV control, to simulate the population dynamics of the DVV. The spatial unit in the Storer [16] model is a 25-hectare field and the whole landscape represents 10 × 10 = 100 fields. Adult dispersal is classified as inter-field dispersal and in-field flight. The time step is a day. Expression of resistance varies from incompletely recessive to incompletely dominant. The functional dominance value has a standard value of 0.1 with a range between 0.0001 and 0.88, depending on the efficacy of the Bt corn trait. The density-dependent mortality of larvae was calculated with a function from Onstad et al. [14]. The model was used to compare the rate at which the adaptation allele spread through the population under different nonresistant corn refuge deployment scenarios, and under various dose levels of the resistance trait. The model output is the relative rate of adaption of the specific scenario, which is the rate of adaption of each run divided by the rate of adaption for the baseline (20% refuge in locations rerandomized each year with default parameter sets). For a given refuge size, the model indicated that placing the nonresistant refuge in a block within a Bt corn field would be likely to delay DVV adaptation rather longer than planting the refuge in separate fields in varying locations. If a portion of the refuge were to be planted in the same fields or in-field blocks each year, DVV adaptation would be delayed substantially. DVV adaptation rate was also predicted to be greatly affected by the level of crop resistance, because of the expectation of dependence of functional dominance on dose. If the dose of the insecticidal protein in the corn is sufficiently high to kill 90% of heterozygotes and 100% of susceptible homozygotes, the trait is predicted to be much more durable than if the dose is lower. A sensitivity analysis showed that parameters relating to adult dispersal affected the rate of pest adaptation. Storer [16] also modeled soil insecticide and crop rotation with different block refuge strategies. Uniform distribution of block refuge and crop rotation is beneficial to delay resistance evolution. 

Onstad et al. [18] expanded a simple model of adult behavior and population genetics to explain how rotation resistance may have developed and to study ways to manage the DVV in a landscape of corn, soybean, and winter wheat. The time step in this model is a generation. The resistance evolution to insecticide or insecticidal trait was not modeled. The model focused on a strategy involving a 2-year rotation of corn and soybean in 85% of the landscape, also evaluated six alternative management strategies over a 15-year time horizon to investigate their effectiveness. Results indicated the rate of evolution increases as the level of rotated landscape increases. The results were most sensitive to increases in the initial allele frequency, the function dominance of the resistance allele and modifications of the density-dependent survival function. 

Crowder and Onstad [17] further expanded the simulation model described in Onstad et al. [18] to study the simultaneous development of resistance to both crop rotation and transgenic corn. The model supports two resistance genes. One gene is for crop rotation resistance and the other is for transgenic corn resistance. The dose of transgenic traits ranged from low dose with 20% survival to high dose with 0% survival. The dominance value of crop rotation resistance was simulated as dominant, additive and recessive. In simulations of areas with rotation-resistant populations, planting transgenic corn to only rotated cornfields (2 year crop rotation) was a robust strategy to prevent resistance to both traits. In areas with rotation resistance risk, planting transgenic corn to only continuous corn fields and planting conventional corn on rotated cornfields was not an effective strategy for preventing adaptation to crop rotation or transgenic corn. In areas without rotation-resistant phenotypes, the gene expression of the allele for resistance to transgenic corn was the most crucial factor affecting the development of resistance to transgenic corn. The model also concluded the initial allele frequency and density dependence were the two most crucial factors affecting the evolution of resistance.

Crowder et al. [28] expanded the deterministic model described in Onstad et al. [14], so the model could evaluate the risk of resistance to both transgenic crops and crop rotation in landscapes with and without rotation-resistant phenotypes as well. This complex model used a daily time-step and had a similar parameter set as Crowder and Onstad [17]. It included daily information of corn phenology, insect phenology, adult survival, emergence, dispersal and mating. 

Results from Crowder et al. [28] indicated that planting transgenic corn to first-year cornfields is a robust strategy to prevent resistance to both crop rotation and transgenic corn in areas where rotation-resistant populations are currently a problem or may be a problem in the future. In areas without rotation-resistant populations, gene expression of the allele for resistance to transgenic corn is the most important factor affecting the evolution of resistance. If expression is recessive, resistance can be delayed longer than 15 year. If expression is dominant, resistance may be difficult to prevent. In a sensitivity analysis, results indicate that density dependence, rotational level in the landscape, and initial allele frequency are the three most important factors affecting the results.

Overall simulation results from a simpler model with a generational time-step [17] compared favorably with a more complex model [28] with a daily time-step. It may be assumed that the complexity involving dispersal, mating, emergence, and oviposition needed to develop the daily model did not affect the results as significantly as other assumptions about gene expression, refuge size, and toxin dose.

Onstad [61] expanded the population dynamics and genetics model published in 2005 by Crowder and Onstad [17] to include larval survival and movement to evaluate the role of mixtures of transgenic and nontransgenic corn seed for resistance management of DVV. The primary concern about seed blends in Onstad [61] was that blends may provide conditions for additional differential selection against susceptibles relative to heterozygotes leading to faster evolution of the pest. The survival of homozygous susceptible individuals (SS), or heterozygotes (RS) with R recessive, is 0, 0.001, 0.05, and 0.20. With R partially recessive, survival of the heterozygotes is 0, 0.01, 0.50, and 0.60 with a theoretical high, practical high, medium, and low toxin dose, respectively. The probability of leaving a nontransgenic plant and the proportion surviving dispersal as neonates are simulated as one of three sets (0.9, 0.06), (0.5, 0.5), and (0.35, 0.95). The probability of leaving a transgenic plant is either 0.9 or 0.5. The survival of first instars due to pre-dispersal tasting of transgenic roots, depends on genotype and allele expression. Onstad [61] studied pre-dispersal tasting survival values of 1 and 0.5 for RR and SS larvae. For heterozygotes, values of 1, 0.75, and 0.55 were simulated respectively. The results for the standard model were not very sensitive to refuge size. The comparison of blocks and seed blends with partially recessive expression was similar. Onstad [61] concluded, given the lack of random mating by DVV inhabiting blocks of transgenic and nontransgenic corn, the seed blends provide a positive effect to the trait durability.

Caprio et al. [19] developed and retrospectively validated a stochastic, individual-based, multifield, simulation model that predicts the evolution of methyl-parathion resistance in DVV populations of Nebraska. The life table parameters of DVV included neonate, larva, pupa, pre-ovipositional adult, young adult and old adult. Resistance to the methyl-parathion aerial adulticide was expressed as a dominant monogenic trait. A single application of methyl-parathion would kill 98.5% of susceptible individuals in the field, whereas 50% of resistant individuals with homozygous or heterozygous genotypes also were killed. Efficacy from a single spray decayed with a half-life of 10 d, and a total period of residual activity equivalent to 20 d. Resistance was determined by the failure of the spray program to reduce beetle populations in the field below a maximum acceptable density of 0.5 gravid females per plant. The landscape in the simulation was a matrix of 25 fields, all treated uniformly. The average plant population per field was 30,000 plants per acre. A consultant survey was conducted to estimate the field control failure of methyl-parathion aerial spray. The population dynamics properties of the model were qualitatively corroborated by comparing the simulated population dynamics with one of several sets of empirical population dynamics published data. Multiple, stepwise regressions were conducted for each of the two resistance measurements to determine model sensitivity to variation in parameters. Bayesian inference was used to estimate the candidate frequency most likely, given reported times to field control failures. Spray timing and multiple spray application strategy were also explored in the simulation. 

The default parameters they incorporated into the model suggested that resistance could have evolved within 8.5 year, while the consultant survey reported control failures at some time with methyl-parathion, averaging 7.6 +/− 1.85 year, after it was initially used. Each decrease of an order of magnitude in the initial resistance allele frequency increased the time until resistance evolved by 2 year. Bayesian inference concluded the initial allele frequency of 10^−4^ was most likely (29%), 10^−3^ was less likely (28%). The model results indicated the time to control failure incorporates both the time it took for resistance to evolve, as well as some time for the population to rebuild to threshold levels. This validated model could also be used to help assess resistance risk for management strategies designed to sustain novel DVV control tactics that may be implemented in the future.

Onstad and Meinke [29] created a simulation model of the population dynamics and genetics of DVV to evaluate the use of refuges in the management of resistance to transgenic insecticidal corn, expressing one or two toxin traits. Hypothetical scenarios and a case study of a corn hybrid pyramided with two common insecticidal traits were simulated. This model extended the work of Crowder and Onstad [17]. The time step for calculation is a single generation (1 year). The main additions to Onstad and Meinke [29] model were (1) a second major gene for resistance, (2) possible reductions in fecundity for survivors of insecticidal corn, and (3) reduction in effective refuge because of limited compliance with refuge requirements. Landscape in Onstad and Meinke [29] is spatially implicit and it is a homogeneous region of cropland consisting only of continuous corn planted with transgenic and nontransgenic hybrids. Two field types were simulated, a field with a block refuge and a block of corn expressing two insecticidal traits, and a field with a block refuge and corn expressing only one insecticidal trait. Functional dominance values for each resistance gene is additive (0.5). The larval survival from density-dependent competition after mortality due to overwintering and toxin exposure was calculated by using a function in Crowder and Onstad [17] and fully explained in Onstad et al. [11]. Onstad and Meinke [29] evaluated required refuge levels of 5, 10, and 20%. The durability of transgenic corn products is defined as the number of years when all resistance allele frequencies reach 0.5. 

In the hypothetical situations, results demonstrated that evolution is generally delayed by pyramids compared with deployment of a single-toxin corn hybrid. However, soil insecticide use in the refuge reduced this delay and quickened the evolution of resistance. Results were sensitive to the degree of male beetle dispersal before mating and to the effectiveness of both toxins in the pyramid. Resistance evolved faster as fecundity increased for survivors of insecticidal corn. Thus, effects on fecundity must be measured to predict which resistance management plans will work well. Evolution of resistance also occurred faster if the survival rate due to exposure to the two toxins was not calculated by multiplication of two independent survival rates (one for each insect gene) but was equivalent to the minimum of the two. Furthermore, when single-trait and pyramided corn hybrids were planted within rootworm-dispersal distance of each other, the toxin traits lost efficacy more quickly than they did in scenarios without single-trait corn. For the case study, the pyramid delayed evolution longer than a single trait corn hybrid and longer than a sequence of toxins based on at least one resistance-allele frequency remaining below 50%. 

Pan et al. [30] created a simulation model of the temporal and spatial dynamics and population genetics of DVV to evaluate the use of block refuges and seed blends in the management of resistance to transgenic insecticidal corn. The model in Pan et al. [30] is based on the work of Onstad et al. [14], Crowder et al. [28], and Onstad [61]. This model combines an adult submodel calculated on a daily time step (adult emergence, dispersal, mating, and oviposition) with a larval submodel calculated once per generation (larval movement and survival). The resistance gene was modeled with the functional dominance value of 0.05. Pan et al. [30] modeled corn rootworm behavior in a set of 80 ha fields planted in corn every year. In the simulation, cornfields are adjacent to each other in a region simulated as a torus; hence, beetles leaving one side of a rectangular region are matched by beetles entering on the opposite side. The 80 ha field was divided into a grid with 10 columns by 8 rows, total 80 cells with each cell of 1 ha. For the fixed-location block refuge, the model chose to place the refuge in the middle of the field. For the scenario in which the refuge is relocated annually within the cornfield, the model alternated the refuge from middle to edge every other year. The daily adult emergence, 7 day protandry and developmental delay of susceptible genotypes were simulated in the adult submodel. In the adult dispersal submodel, the dispersal distance follows an exponential distribution and the dispersal direction is random. The male dispersal was modeled as a mean dispersal rate of 15 m per day and sensitivity analysis varied the mean dispersal rate from 5 to 45 m per day. The mated female adults disperse at a mean dispersal rate of 15 m per day, the same as male. Furthermore, the influence of more frequent random-walking with constant dispersal distance per step was also modeled. In the larval submodel, overwintering egg survival, larval movement and survival was modeled. The density-dependent survival occurs after mortality due to overwintering and Bt corn root exposure was adjusted by using a function in Onstad [61]. 

Pan et al. [30] concluded that the seed-blend scenarios in many cases produced equal or greater durability than block refuges that were relocated each year. The standard analysis presumed complete adoption of Bt corn by all farmers in the region, no crop rotation, and 100% compliance with IRM regulations. As compliance levels declined, resistance evolved faster when block refuges were deployed. Seed treatments that killed the pest when applied to all seeds in a seed blend or just to seeds in Bt corn blocks delayed evolution of resistance.

Caprio and Glaser [35] developed two simulation models to evaluate the DVV resistance evolution and management. The two models consisted of a modification of a spatially-explicit, stochastic model POPGEN-S2 [19] and a simpler, frequency-based deterministic model, POPGEN-D. The latter could be run in a single simulation mode with a graphical interface to enter parameters or in a risk-assessment mode capable of running thousands of simulations to estimate the effects of parameter uncertainty. 

The biological parameters of the stochastic model were primarily derived from Caprio’s previous DVV model [19] except for mortality rates, development delay, block size and dispersal rates. The stochastic model in Caprio and Glaser [35] was used only for evaluations of four different block refuge scenarios. The functional dominance values of 0.05 and 0.2 were simulated in the stochastic model. The simulation landscape consisted of a 40 × 40 matrix of 30 m × 30 m blocks, total 144 ha. Four different field sizes were simulated as 5 × 5 blocks, 10 × 10 blocks, 20 × 20 blocks and 40 × 40 blocks. Refuge was annually planted on one edge or opposite edge of each field randomly. Adult dispersal was modeled with the movement of an average of 41 m/day. Movement was assumed as a Brownian diffusion-based random walk. Additional rates for mortality and developmental delay of larvae on transgenic plants were adopted from a model, submitted to USEPA for product registration (DuPont Pioneer unpublished data). The stochastic model indicated the field size was highly significant to determine the resistance development. The results also indicated that rather than nonrandom mating being the threat to a block refuge system, it is nonrandom oviposition and refuge placement that are most important.

The deterministic model is a simple frequency-based model that incorporates noncompliance, nonrandom mating in compliant fields and larval movement [35]. It can simulate block refuge and blended refuge. The parameters in the deterministic model were drawn from uncertainty distributions, Pert distributions characterized by estimates of the most likely, minimum and maximum values. While each run of the simulation was deterministic, 1000 runs were conducted, each with randomly drawn parameters from the relevant uncertainty distributions. One of the key parameters in the deterministic model is the effective refuge size of block refuge. The worst-case effective refuge sizes for the four different field sizes were (from smallest to largest field size): 15%, 11%, 3% and 0.055% when the refuge was planted in the area of the field where the egg density was the lowest. For blended refuge, larval movement composed of base larval movement and asymmetrical movement, which accounts for larvae tending to taste and move from transgenic tissue at a greater rate than on non-Bt tissue. The movement rate is varied from 0% to 50%, with 30% as the most likely value. This model supports two loci and can compare the relative longevity of two genes stacked into the same plant versus using the same two genes sequentially in single gene plants. 

Caprio and Glaser [35] concluded the durability of the block refuge is very sensitive to field size and the block refuge is better than the blended refuge at delaying resistance in the simulation of the deterministic model. The deterministic model is a frequency-based model and does not support population information, so it does not incorporate density dependent survival. This might overestimate the longevity of 20% refuge as the larval density is high enough to trigger density dependent mortality. Model results also indicated the expected lifetime of the dual gene pyramid product is much longer than if the two toxins had been used sequentially regardless of block or blend refuge deployment.

Kang et al. [36] created a daily time step population genetics model to study the effects of temporal separation between male and female beetle emergence from Bt and non-Bt corn and a female-skewed sex ratio for DVV emerging from Bt corn on the evolution of Bt resistance. The model simulated a 100 ha landscape with 20% blended refuge for single-trait Bt corn and 5% blended refuge for pyramided Bt corn. Two unlinked, loci in DVV were assumed to determine resistance to transgenic corn expressing Bt toxins. The toxin mortality was applied before density-dependent mortality occurred. The standard survival value for homozygous susceptible items was set at 0.01, and the standard dominance was set to 0.5. The density-dependent mortality occurred after toxin mortality. Variant sex-specific toxin mortality was assumed to determine the sex ratio of beetles emerging from Bt corn.

The standard values of male and female developmental delay were 0–3 and 0–2 days, the average of the differences between the mean emergence time for beetles from transgenic corn and the non-Bt corn. The sensitivity analyses ranged from 0 to 28 days. The effect of great emergence delays (>14 days) on resistance evolution was studied for theoretical purposes. 

Kang et al. [36] concluded the effect of skewed toxin mortality in one sex on evolution of Bt resistance was insignificant. An emergence delay among resistant beetles from Bt corn slowed resistance evolution. A shift in the time of emergence for homozygous susceptible beetles from Bt corn did not have a significant effect on the evolution of Bt resistance in DVV. 

Adult emergence delay has been a critical issue in DVV resistance management. Onstad et al. [14] simulated an emergence delay for homozygous susceptible beetles of 3–9 d in transgenic corn in fields with row strip or block refuges. They concluded that the emergence delay by susceptible beetles and the configuration of the refuge significantly accelerated the evolution of resistance by DVV. Storer [16] included developmental delays for susceptible and heterozygous beetles. For susceptible homozygotes and heterozygotes surviving on transgenic corn, the delay is 7 d and 3.5 d respectively. Caprio and Glaser [35] modeled 5% larvae feeding on transgenic corn were held back from each age group (though they did experience mortality) and did not age. This resulted in a mean developmental delay of 2.3 days. Pan et al. [30] included a 7-d delay in emergence for susceptible adults and heterozygotes emerging in transgenic corn compared with those insects emerging in refuge while there is no developmental delay for resistant adults. They showed that this type of developmental delay slightly delayed the evolution of Bt resistance by DVV. Kang et al. [36] modeled developmental delays in seed blends. The standard developmental delays for males and females were 3 d and 2 d and the sensitivity analysis evaluated 0 to 28 d. The results from Kang et al. [36] supported Pan et al. [30].

Martinez and Caprio [32] developed a two-gene, discrete generational and spatially explicit model with a probabilistic and deterministic mode for DVV. The model consisted of six separate, successive developmental and behavioral phases that occurred once per year: egg stage, larval stage, density-dependent mortality, pre-mating adult dispersal followed by mating and post-mating dispersal. This model is used to investigate the effects of IPM strategies along with planting of a structured refuge or seed blend on the lifetime of a hypothetical nonhigh dose Bt toxin pyramid.

The landscape consisted of a 51 × 51 field matrix (one field represented 50 ha (707 × 707 m) of corn with 4 million plants; matrix size = 36.1 km × 36.1 km) and was designed as a torus. A two-dimensional Gaussian redistribution kernel was used to simulate adult movement through space under the assumption of Brownian motion. The diffusivity parameter D (field^2^/time step) describes the pest’s propensity to disperse. Based on the daily dispersal rate 29 m/day and 775 m for lifetime dispersal, the diffusivity D was chosen as 2 field^2^/generation for pre-reproductive adult dispersal and 4 field^2^/generation for post-mating female dispersal. The model had only 10% adults perform pre-mating dispersal and 15% female performed long-distance post-mating dispersal. 

Density dependent survival in the model occurred after natural and *Bt* mortality took place and was simulated as scramble competition using the Hassell equation to regulate population densities on refuge and Bt plants (separately) in each field. More details of the density dependent survival under scramble competition can be found in Martinez et al. [23]. The parameters were adjusted to result in an overall egg-to-larval survival of 4.5% in refuge under standard parameter set. The oviposition was assumed to be uniformly distributed across the 50-ha fields (blocks and fields with blended seed). Base larval movement in blended refuge was set to 30%.

Martinez and Caprio [32] concluded that crop rotation was the most effective strategy, followed by increasing the non-Bt refuge size from 5 to 20%. Soil applied insecticide use for Bt corn did not increase the durability compared with planting Bt with refuges alone, and both projected lower durabilities. The mitigation with random selection of strategies was ineffective at slowing resistance, unless crop rotation occurred immediately; regional mitigation was superior to random mitigation in the area with high levels of resistance and reduced the observed resistance allele frequencies in the neighborhood.

## 6. Models of *Diabrotica* Geographic Spread (>1 km/Year for Multiple Years)

*D. virgifera virgifera* (DVV) can fly several km using its own power and have flight distance lengthened by wind and storms. It is also possible that long-distance movement can be assisted by human activity. This section describes the wide variety of models that have been used to explain or predict geographic spread.

Onstad et al. [63] created and analyzed a set of simple meteorological and behavioral models that can be used to predict the spread of populations of DVV that had lost fidelity to corn, and therefore, commonly infested soybean (*Glycine max*) throughout the north central United States starting in the 1990s. These crop rotation-resistant beetles spread outwards from a focus in Illinois to infest multiple states over several years. This work extended the modeling performed by Onstad et al. [64] to determine how far and in what directions the population would spread in Illinois, Indiana, Michigan, and Ohio. Their primary hypothesis was that increased landscape diversity slows the rate of regional spread of the rotation-resistant DVV over several years. Thus, some of the models invoked a landscape-diversity function that included the proportion of noncorn, nonrotated-soybean vegetation on farmland in each county [63,64] called this extra vegetation). 

Using logic and some flight mill data [43], Onstad et al. [63] determined that DVV adults can fly 3.9 km without wind support during the 1.30 h available on a typical summer day (based on temperature). Because the wave front of beetle dispersal will be determined by those flying downwind, the mean wind speeds for the 18 directions were calculated from the climate data. From these and the logic of the number of hours of flight per day, Onstad et al. [63] calculated the maximum possible wind-supported spread for each direction.

They used summer data for temperature, wind speed, and direction for several north central states to calculate wind-supported movement. In addition, to determine the role of storms, Onstad et al. [63] used published observations of heavy rainstorms and heavy rain cells (i.e., areas through which rain passes) to calculate probability distributions for storm tracks typical for the north central United States. Most movement is eastward or northeastward (coming from the west) with a maximum distance of 33 km [64]. This wave front rate was hypothesized to be valid for any threshold of measurement used to define the spread of the DVV. One sector (direction) was allowed maximum distance of spread; all others had reduced distances based on the proportion of storms observed for a given sector. Dispersal out of each sector was given the maximum of either wind-supported or storm-supported movement.

Finally, Onstad et al. [63] used data on the amount of farmland that was not corn or rotated soybean to calculate the proportional area of extra vegetation, which will act as a sink for the spreading population because offspring will die in fields without corn roots. Onstad et al. [63] used this information for each county to adjust the quality of each 1 km^2^ cell. Their standard model used a linear effect to adjust the radii of dispersal used for each cell, depending on which of eight categories of extra vegetation that cell was in. For all cells within each category, the maximum radius for each sector was multiplied by (1-MEV), where MEV represents the mean value of extra vegetation for that category. They simulated the model for 16 years from 1986 to 2001 using geographic information system software to determine the presence of rotation-resistant DVV in grid cells in several states. The results were tested with observations from 1997–2001.

The best model for the period from 1997 to 2001 is based on heavy-storm data, with distance that beetles spread each year reduced by the proportion of extra vegetation in a county [63]. This model was superior to the previously published model and to two other models that do not consider landscape diversity. Most of the models predicted spread at too high a rate between 1997 and 2001, compared with observations, but a few new models with rates of spread reduced by a landscape-diversity function matched the observations relatively well. 

Hemerik et al. [65] combined a temperature-based population dynamics model with a dispersal model [66] to predict how temperature differences would influence range expansion after the year 2000 in Europe, the initial year in the simulations. They created a mechanistic model of DVV maturation and population growth based on information in the literature. Adult flight behavior was estimated from flight mill studies of Coats et al. [43]. Hemerik et al. [65] also used literature information to calculate net reproduction rate based on temperature and then calculate velocity of range expansion, which they considered to be a random process.

Based on observations made from 1992–2000 in Europe, Hemerick et al. [65] determined that the mean velocity of range expansion was 33 km/year. Hemerik et al. [65] found that their simpler model underestimated the velocity of range expansion compared to observations, whereas their more-detailed model overestimated the same. They concluded that reliance on laboratory data to predict dynamics of field populations is a major problem. 

Carrasco et al. [47] created and tested a model that incorporated natural and human-assisted long-distance dispersal of DVV in Austria. Their purpose was to determine the most important mechanisms explaining the observed pattern of spread of the DVV invasion in Austria. They also used the model to evaluate how effective the European regional measures for stopping the spread of DVV were likely to be. DVV was first detected in Austria in 2002–2003.

Human-assisted dispersal of DVV was believed to be relevant because isolated outbreaks of DVV occurred far beyond the main body of the invasion near important Danube River harbors and along highways. Thus, the model accounted for highways, railways and rivers. The Danube River basin is especially relevant because it is one of the most common means of international transport in Europe. Furthermore, farmers tend to grow continuous corn along the riverbanks. 

The model by Carrasco et al. [47] calculated within-field population dynamics and various types of dispersal. Patches were cornfields and “macro-fields” which represented the area of corn grown in each municipality as a single field. Altitude or elevation of fields was considered in the model. For natural dispersal, they used two alternative probability distributions to calculate radial distance for a given direction. These probabilities were influenced by field size and growth stage of the corn crop.

Carrasco et al. [7] considered two types of human-assisted, long-distance dispersal. Cities were modelled as attractors drawing some DVV away from farmland. DVV adults within one day flight to the transportation lines connecting the 10 largest cities in Austria could move long-distances towards these cities. Human-assisted dispersal related to international transport through the Danube River basin was modeled by assuming that DVV adults within one day flight of the Danube River could also move long distances. Both of these types of dispersal included distances based on a negative power law dispersal kernel.

Carrasco et al. [47] calibrated the model by using maximum likelihood techniques combined with spatial stochastic simulation to fit model results to observed data from the historical invasion of Austria in 2002–2004. They then tested the model predictions against independent observations from 2005–2008.

Carrasco et al. [47] found that the model that included the human-assisted, long-distance dispersal through the Danube River basin matched the independent data set the best. In fact, results of this model had a very good fit between predictions and observations. Other models that only considered natural dispersal or only the influence of cities on dispersal were inadequate. Therefore, the authors concluded that the spatial pattern of the invasion could only be explained by a combination of natural dispersal and human-assisted dispersal along the Danube River. 

To facilitate European Pest Risk Analyses, Robinet et al. [67] developed two generic spread models that consist of a set of submodels for population dynamics and dispersal, with linkages to niche maps that are based on climate and habitat suitability. They used the outputs of a CLIMEX model [68] run with climate data to define the area of potential establishment.

One model determines occupancy of cells, not density of DVV. It simulates radial range expansion at a constant rate, similar to reaction diffusion models. It has a single parameter, the constant expansion rate, which accounts for distance of dispersal and population growth. The rate is the same in all directions. All cells within the circular predicted range are considered invaded. For DVV, Robinet et al. [67] used a constant radial rate of expansion of 80 km/year.

The other model of Robinet et al. [67] calculates population density. The population growth process is logistic with two parameters: carrying capacity and growth rate. They modeled dispersal using a t-distribution with two parameters: a length scale and a shape parameter. They recommend the rotated t-distribution for its versatility in studies of geographic spread. The fatness of the tails, which reflects the likelihood of long-distance dispersal events, is determined by the shape parameter. A kernel with fat tails may be used to represent a situation in which human activity, for example, can be responsible for occasional spread events over much longer distances than are attained by biological spread mechanisms. Unfortunately, the shape parameter is very difficult to measure and is almost impossible to estimate from dispersal data according to Robinet et al. [67]. Robinet et al. [67] used a shape parameter that formed a fat-tailed dispersal kernel and caused a large proportion of DVV to disperse over long distances within Europe. They assumed the same radial expansion rate as in their first model (length scale = 80 km).

Robinet et al. [67] concluded that the key difficulty in using these simple models is estimating the parameters. For DVV, values of parameters are not directly published in the literature, and a good understanding of the parameters’ meanings in the context of the model are needed to extract the required values from data. They also concluded that, for their density-dispersal model, the shape parameter governing the proportion of long-distance dispersers does not have the strongest effect on the predicted area invaded. This is the opposite of what Carrasco et al. [47] concluded. 

Ecological site occupancy models used climate data combined with DVV distribution data to predict whether a given site is likely to be occupied (or capable of being occupied) by DVV. These have been particularly important in areas such as Europe where DVV is a relatively recent invasive species to determine the regions where DVV is likely to become established. These models can also be important when attempting to predict distribution shifts due to climate change. Aragon and Lobo [69] used a multidimensional envelope model customized for DVV while Bernardi et al. [70] used a geographic information system and agroclimate data to estimate possible European distributions. Eitsinger et al. [71] expanded this to include soil temperature and ground cover data to estimate lower developmental temperature thresholds. Dupin et al. [72] developed a training set of presence/absence data that they combined with 19 climatic features and estimated DVV distribution using several envelope models as well as support vector machine, a supervised machine learning technique. In this case the envelope models, with more domain-specific knowledge, outperformed the support vector machine, though all had significant misclassifications. Grozea et al. [73] used trap captures and climate data in Romania to predict presence and abundance. Feature or variable selection can be difficult when developing site occupancy models as significant collinearity can exist [74]. More conventional models can be combined with these climate-based models. For example, a degree-day driven model was combined with global warming estimates to estimate distribution shifts of both DB and DVV [75], warning that DVV could “become dominant in new areas such as northern Minnesota and the Dakotas”.

## 7. Discussion

We believe that model simulation is essentially virtual experimentation [3,33,76]. Such “in silico” models are often used when experiments on the entire system, such as world climate or resistance to toxins, are either infeasible or ethically challenging. 

The biological data and processes included in any model depend upon its intended use. The modeling goal helps modelers focus on the real ecological system and sharpens the focus on the major components of interest. Some models can be quite simple with only a few parameters. However, in some cases, these parameters are actually meta-parameters, summarizing many underlying parameters, such as the exponential growth rate for a population. A population’s generational growth rate, for example, would encompass at the very least fecundity, natural mortality, overwintering survivorship and perhaps diffusion as well. 

Onstad [77] noted that every model of the same system has the same total number of implicit and explicit assumptions. For example, a density-based, population-genetics model provides the ratio of male and female offspring and explicitly calculates both male and female adults. However, an allele-frequency based model would not provide explicit information about males and females and therefore would need to make implicit assumptions about them. In general, if one model has a mathematical function (explicit assumption) for a process or component, then a different model of the same ecological system, but without that function, can be considered to have one more implicit assumption regarding the system. 

The eminent statistician GeorgeE. Box [78] noted that “all models are wrong, but some are useful”. While the implications of this statement can be debated, it is clear that Box preferred the most parsimonious model possible, favoring simple models over more complex models if simple models can draw the same conclusion as complex models. In a tribute to RonaldA. Fisher [79], Box stated that the modeler “… should seek an economical description of natural phenomena. Just as the ability to devise simple but evocative models is the signature of the great scientist so overelaboration and over-parameterization is often the mark of mediocrity”. Onstad [80] addressed the issues of simplicity, generality, realism and precision. He concluded that generality is not a property of a model that can be identified nor proclaimed at the time of a model’s creation. A model is determined to be general after it has been tested against many systems. A model’s accuracy and precision can be evaluated when it is tested against independent data. Complex and simple models can produce the same degree of accuracy and precision. Some complex models can also estimate, through stochastic variability, variance components beyond those identified through sensitivity analysis. Nor are these two approaches exclusive of each other. Occasionally, in mathematical models, we have found unexpected results in complex models and using those insights, developed simplified models to explore the same process in a less complicated environment [42].

Onstad [77] suggested that models should be made as realistic and as simple as possible to achieve the goal. A model that permits all individuals to reproduce without accounting for the differences between males and females or between immatures and adults cannot be considered as real as a model that has more realistic reproduction. For example, exponential growth of a population may seem to work fine at some gross scales of time, but the same function cannot be used when only immatures are alive over a given period.

The modeling purpose determines the complexity of the models, so perhaps it is not surprising that the reviewed models for DVV IRM tended to have similar model structures. Most were deterministic, but some involved stochastic processes. The genes resistant to transgenic traits and chemical compounds were assumed to have unlinked, diallelic autosomal loci. Onstad et al. [14] and Storer [16] provided guidelines for DVV models and had a complete list of DVV biological parameters. Although they used very different durability definitions, critical parameters to determine the durability were the same. Onstad et al. [18,63] Modeled rotation resistance and concluded high rotation adoption is a key factor to drive resistance. Crowder and Onstad [17] and Crowder et al. [28] extended this model approach and incorporated transgenic corn and crop rotation into the crop system. Onstad [61] modeled blended refuge and concluded larval movement might not decrease the durability of blended refuge significantly and blended refuge could overcome the less than complete compliance with block refuge requirements. Caprio et al. [19] used a model to retrospectively validate the evolution of resistance to adulticidal sprays of methyl-parathion. This model was expanded by Caprio and Grasser [35] to evaluate block refuge effectiveness with different field sizes. The model concluded that block refuge is less effective when the field size increases. Onstad and Meinke [29] expanded on the Crowder and Onstad [28] model and evaluated the resistance to single trait and pyramided transgenic corn. They concluded that novel traits are better than existing commercial traits when pyramids are commercialized. The benefit decreases further if survival from the pyramid is the minimum survival on either single trait instead of the product of survival of each trait. The modeling by Pan et al. [30] indicated that blended refuge produced greater durability than block refuges when the blocks either had compliance issues or were relocated each year due to nonuniform oviposition, which was consistent with findings in Caprio and Glasser [35]. Kang et al. [36] modeled temporal separation between male and female beetle emergence due to developmental delay on Bt corn and female-skewed sex ratio for adults emerging from Bt corn. They concluded the effects of these two factors on trait durability were insignificant. Martinez and Caprio [32] simulated different IPM strategies that could support IRM plans for DVV. They concluded that crop rotation was the most effective strategy and soil applied insecticide used for Bt corn would not help to boost the durability. Regional mitigation was a better strategy than random mitigation in an area with high levels of resistance. 

It is noteworthy that, in spite of the difficulties of quantitatively studying insects that spend their immature stages underground and have only one generation per year with obligate diapause, scientists have found many reasons and many ways to model the *Diabrotica* described here. All resistance management models we reviewed are enriched with detailed demography and population genetics, which significantly impact model results. Other general models ignoring population biology might not be suitable for predicting evolution of resistance. The models of geographic spread demonstrate that modeling can involve many approaches and styles. We are inspired by the intellectual challenges these pests provide and the creative responses by the scientists who model them.

## 8. Conclusions

Many papers were reviewed and found to be valuable for guiding research in the future. Modeling is important because both of these species are difficult to manage.

## Figures and Tables

**Table 1:** Purpose of model relative to integrated pest management (IPM) determines the complexity of model structure.

Reference	Time Step	Larval Movement	Adult Dispersal	Simulated Landscape	Purpose of Model
Onstad et al. [14]	Day	No	Cross patches	Spatially implicit, 100 ha with patches of corn and soybean	Estimate durability of rootworm traits with various structured refuge proportion
Storer [16]	Day	No	Intra-field and inter-field	Spatially explicit, 100 fields with each field of 25 ha planting corn or soybean	Estimate durability of rootworm traits with various structured refuge proportion
Onstad et al. [18]	Generation	No	Cross patches	Spatially implicit, 100 ha with patches of corn and soybean	Rotation resistance
Crowder and Onstad [17]	Generation	No	Cross patches	Spatially implicit, 100 ha with patches of corn and soybean	Transgenic resistance and rotation resistance
Crowder et al. [28]	Day	No	Cross patches	Spatially implicit, 100 ha with patches of corn and soybean	Transgenic resistance and rotation resistance
Onstad [61]	Generation	Yes	Cross patches	Spatially implicit, 100 ha with patches of corn and soybean	Comparison of blended refuge vs. block refuge
Caprio et al. [19]	Day	No	Cross patches	Spatially explicit, matrix of 25 fields	Retrospectively validated evolution of methyl-parathion resistance
Onstad and Meinke [29]	Generation	No	Cross patches	Spatially implicit, 100 ha with patches of corn and soybean	Durability of pyramid
Pan et al. [30]	Generation for larval stage and day for adult	Yes	Intra-field	Spatially explicit, 80 ha field gridded with 1 ha cells	Comparison of blended refuge vs. block refuge
Caprio and Glaser [35]	Day	No	Intra-field and long-range dispersal	Spatially explicit, multiple fields with four different field sizes	Evaluate block refuge effectiveness
Kang et al. [36]	Day	No	Ignored	Spatially implicit, seed blend in 100 ha field	Evaluate emergence delay and skewed sex ratio
Martinez and Caprio [32]	Day	Yes	Inter-field	Spatially explicit, 51 × 51 field matrix with 50 ha field	Evaluate durability benefit of IPM strategies to transgenic corn
